# Left Ventral Caudate Functional Connectivity Mediates the Relationship Between Habitual Responding and Alcohol Use

**DOI:** 10.1111/ejn.70150

**Published:** 2025-05-26

**Authors:** Lindsey R. Fisher‐Fox, Mario Dzemidzic, McKenzie R. Cox, David Haines, James Hays, Mayande K. Mlungwana, Zachary Whitt, Andrea Avena‐Koenigsberger, Ann E. K. Kosobud, David A. Kareken, Sean O'Connor, Martin H. Plawecki, Melissa A. Cyders

**Affiliations:** ^1^ Department of Psychology Indiana University Indianapolis Indianapolis USA; ^2^ Department of Neurology Indiana University School of Medicine Indianapolis USA; ^3^ Department of Psychiatry Indiana University School of Medicine Indianapolis USA

**Keywords:** functional magnetic resonance imaging, habitual behaviour, human, intravenous alcohol self‐administration, resting state

## Abstract

Preclinical studies posit that habitual behaviour is an important mechanism in the development of alcohol use disorder (AUD), but human findings are unclear. The goals of this study were to test a behavioural measure of habit formation, the Slips of Action Task (SOAT), in humans and identify brain‐based mechanisms explaining the relationship between habit and alcohol use. Thirty‐six participants (63.9% female, mean age = 30.58, SD = 9.73, 69.4% White, 83.3% Not Hispanic/Latino) who endorsed heavy drinking completed self‐report measures, the SOAT (lower scores = higher habit formation), a 2.5‐h intravenous alcohol self‐administration session, and a resting‐state functional magnetic resonance imaging scan. Three seed regions—bilateral ventral caudate, nucleus accumbens and dorsal caudate—were assessed for significant whole brain functional connectivity (FC) associations with SOAT (cluster‐level *p*_*FWE*_ < 0.05 at a cluster‐forming threshold *p* = 0.001). Two clusters survived Bonferroni correction (cluster *p*_*FWE*_ = 0.008): FC between the left ventral caudate and the left middle frontal gyrus correlated negatively, while FC between the left NAc and the right central operculum correlated positively, with SOAT score. SOAT score was unrelated to drinking outcomes; however, there was a significant indirect relationship between SOAT and average drinks per drinking day through FC between the left ventral caudate and the left middle frontal gyrus. A similar trend seen with cumulative work for alcohol fell short of significance. Habit formation's relationship with alcohol use may function through neuroadaptations in the ventral caudate. More work is needed to better characterize objective habit formation in the human alcohol laboratory with additional laboratory‐, alcohol‐specific, imaging‐ and ambulatory‐based alcohol use metrics.

## Graphical abstract

Resting state functional connectivity (FC) between the left ventral caudate and the left middle frontal gyrus (MFG) correlated negatively, while FC between the left nucleus accumbens (NAc) and the right central operculum (CO) correlated positively, with Slips of Action Task (SOAT) score. The relationship between SOAT and average drinks per drinking day was significantly mediated by FC strength of the left ventral caudate and left MFG. A similar trend with cumulative work for alcohol fell short of significance. Solid arrow indicates a significant association. Dashed arrow indicates nonsignificant association.

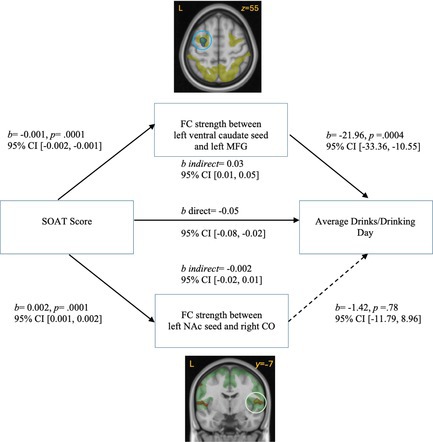

**Abbreviations**
ANTs: advanced normalization tools
AUD: alcohol use disorder
AUDIT: alcohol use disorder identification test
BOLD: blood oxygenation level dependent
BrAC: breath alcohol concentration
CAIS: computer‐assisted infusion system
CAT: constant attention task
CO: central operculum
EPI: echo‐planar imaging
FC: functional connectivity
FoV: field‐of‐view
FWE: family‐wise error
FWHM: full width at half maximum
GRAPPA: GeneRalized Autocalibrating Partially Parallel Acquisitions
HCP: human connectome project
ICA: independent components analysis
IV: intravenous
MFG: middle frontal gyrus
MB: multi‐band
MRI: magnetic resonance imaging
MNI: Montreal Neurological Institute
NAc: nucleus accumbens
ROI: regions of interest
rsFC: resting state functional connectivity
SD: standard deviation
SPM: statistical parametric mapping
SPSS: statistical product and service solutions
SOAT: Slips of Action Task
TR/TE/TI: repetition/echo/inversion time

## 1 Introduction

Habitual behaviour is an important mechanism leading to the development of alcohol use disorder (AUD) (Belin‐Rauscent et al. 2012; Corbit and Janak 2016; Everitt and Robbins 2005; Ostlund and Balleine 2008). Habitual behaviour is commonly modelled preclinically with reward devaluation of alcohol reinforcement (Dickinson et al. 2002; Mangieri et al. 2012), alcohol acclimation (Corbit et al. 2012) and free choice drinking paradigms (Corbit et al. 2012; Cuzon Carlson et al. 2011; Dickinson et al. 2002; Mangieri et al. 2012); it has also been targeted for pharmacological intervention (Hay et al. 2013; Witkiewitz et al. 2019). Most human studies assess habit formation indirectly, relying on self‐reports of alcohol‐seeking as a proxy for a more general proclivity toward habit formation (Piquet‐Pessôa et al. 2019; Ray et al. 2020; Wyckmans et al. 2020). The goal of the current study was to conduct a test of the Slips of Action Task (SOAT) (De Wit et al. 2007), a behavioural measure of habit formation, as related to alcohol‐seeking in the human laboratory and to identify brain‐based mechanisms explaining the relationship between SOAT performance and alcohol use.

The Dual Systems Model (Casey et al. 2008; Steinberg 2017) proposes that behaviour is governed by a balance between two competing systems: a goal‐directed system and a habitual system. Goal‐directed behaviours require relatively more cognitive resources, but they are flexible and can be modified when action‐outcome contingencies change, whereas habitual behaviours are more resource‐efficient, but less behaviourally flexible, and may be maintained even when outcomes are maladaptive. Although AUD is thought to reflect a shift from goal‐directed alcohol‐seeking to habitual use (Koob and Volkow 2010), the data in human studies are mixed: a recent review of nine human studies (Giannone et al. 2024) concluded that although evidence suggests that AUD is associated with reduced goal‐directed, rather than increased habitual behaviours (Sebold et al. 2014), this has not always been replicated (Nebe et al. 2018; Sebold et al. 2017; Voon et al. 2015).

Goal‐directed and habitual behaviours are encoded in the brain through distinct neural pathways (McClure and Bickel 2014; McKim et al. 2016). In rodents, the striatum, especially the ventral striatum, is involved in reward‐based learning for goal directed actions. Over time, control shifts to the dorsal/dorsolateral striatum, particularly the caudate and putamen (Smith and Graybiel 2013; Yin et al. 2004, 2006), which, when combined with reduced innervation from the prefrontal cortex to regions such as the ventral striatum and the nucleus accumbens (NAc) (Voorn et al. 2004), contributes to the transition from goal‐directed to habitual responding. In humans, the shift from prefrontal to dorsal striatal control marks the shift from flexible, goal‐directed actions to automatic, habitual responses, although effects may vary across caudate subdivisions. The dorsolateral striatum encodes behavioural automaticity (Lipton et al. 2019), while the ventral caudate is linked with reward anticipation, incentive motivation and action (Castro and Bruchas 2019; Pauli et al. 2016). Activation of the ventral caudate leads to the reinforcing of goal‐directed motor sequences mediated by the NAc and prefrontal cortex (Fattore and Diana 2016), which encode stimuli value and action outcomes, respectively (Mannella et al. 2013). Thus, discrete contributions from the ventral caudate, dorsal caudate and NAc may underlie the shift to habitual responding in humans.

The SOAT measures the balance between the habitual system and the goal‐directed system in humans, using an instrumental learning paradigm (De Wit et al. 2007) that is not subject to self‐report biases and may offer more clarity as to the balance between goal‐directed and habitual responding in AUD. The paradigm begins with instrumental discrimination training where participants learn to associate correct responses (i.e., left or right key presses) with six different stimuli. Subsequent phases assess the degree to which they formed stimulus → response habitual associations or outcome → response goal‐directed associations. Since the test notifies participants of outcome devaluation, the formation of outcome → response associations results in better performance. Responses to devalued outcomes are considered habitual and indicate an overreliance on habits in instructional control, whereas responses to valued outcomes are considered to reflect behavioural flexibility and goal reliance. Goal‐ and habitual‐responding differences on the SOAT are associated with alcohol dependence (Sjoerds et al. 2013), cocaine use disorder (Ersche et al. 2016) and smoking dependence (Luijten et al. 2020).

Identifying neural correlates of the SOAT offers the advantage of distinguishing between deficits in the goal‐directed system or enhancement of the habitual system to better explain alcohol use behaviour (Watson et al. 2018). SOAT goal‐directed behaviour correlates with ventromedial prefrontal‐caudate connections, whereas habitual responding relates to premotor–posterior putamen connections and higher grey matter density in the posterior putamen (de Wit et al. 2012; van Timmeren et al. 2024). In AUD, SOAT habitual learning associates with increased activation in the posterior putamen and dorsal caudate, with decreased activation in the ventromedial prefrontal cortex and anterior putamen (Sjoerds et al. 2013). SOAT habitual and goal‐directed actions correlate with greater activity in the premotor cortex and caudate, respectively (Watson et al. 2018). Impulsive reward anticipation responses correspond with activation in the dorsal anterior cingulate cortex, inferior frontal cortex, parietal operculum, striatum and thalamus in healthy controls, stimulant dependent individuals and those with a sibling with stimulant dependence (Zhukovsky et al. 2020). Response conflict during SOAT shows increased activation in the anterior cingulate cortex, paracingulate gyrus, lateral orbitofrontal cortex and inferior frontal gyrus, while overcoming response conflict involves the caudate and dorsolateral prefrontal cortex (Watson et al. 2018).

The goal of this study was to examine how SOAT corresponds with alcohol‐seeking and use, and to identify brain‐based mechanisms of this relationship, extending previous literature by including resting state cerebral functional connectivity (FC) and a direct measure of alcohol‐seeking. We hypothesized, supported by previous literature, the following:

Hypothesis 1 *SOAT score would correlate with average drinks per drinking day, reflecting habitual alcohol use, and work completed for alcohol in the laboratory, reflecting motivation for alcohol*.

Hypothesis 2 *SOAT score would relate to brain FC with bilateral seed regions in the ventral caudate, dorsal caudate and NAc*.

Hypothesis 3 *The relationship between SOAT and alcohol use would be mediated by FC with these seed regions*.

## 2 Materials and Methods

### 2.1 Participants

Participants were 36 adults (mean age = 30.58, SD = 9.73, 63.9% female, 69.4% White, 83.3% Not Hispanic/Latino) who were recruited from the general community as part of a larger project (P60AA007611). Participants were recruited to ensure both a range of lifetime drinking history and, for safety, sufficient recent experience with alcohol's effects. Inclusion criteria included good health (as determined by study physician via self‐report and medical history), BMI > 18.5 kg/m^2^, aged 21–55 and able to understand/complete questionnaires and procedures in English. Additional inclusion criteria were the following: good health (assessed via self‐reported medical conditions), screening labs (e.g., liver function tests), right‐handed, vital signs assessment, review of medications and screening measures), aged 21–55, able to understand/complete questionnaires and procedures in English and venous access sufficient to allow blood sampling. Exclusion criteria were the following: pregnant or breast‐feeding, desire to be treated for any substance use disorder or court ordered not to drink alcohol, medical or mental health conditions or medications that may influence data quality or participant safety, and positive urine drug screen (for amphetamines/methamphetamines, barbiturates, benzodiazepines, cocaine, opiates or phencyclidine) or breath alcohol reading on arrival on any study day precluding study completion. Participants of the larger project who completed all study sessions, including an optional imaging assessment, and in which the SOAT was collected, were included in the current analyses. One participant was excluded due to being left‐handed.

### 2.2 Measures

#### 2.2.1 Demographics

Participants self‐reported age, biological sex, race and ethnicity.

#### 2.2.2 The SOAT (De Wit et al. 2007)

A computerized task based on the SOAT was created, consisting of four phases: training, outcome devaluation, knowledge test and slips of action. Training teaches participants to associate correct responses (i.e., left or right key presses) with six different visual stimuli. During outcome devaluation, some of the previous reward images become worthless and earn no points (denoted with an X over the image). This phase assesses the degree to which participants formed S (stimulus) → R (response) habitual associations or O (outcome) → R goal‐directed associations. Since the test notifies participants of outcome devaluation, the formation of O → R associations would result in better performance. The Knowledge phase explicitly assesses response and outcome knowledge of each stimulus. Finally, the SOA phase provides a sensitive index of the balance between goal‐directed vs. habitual control; participants are presented with a rapid succession of stimuli and are asked to respond when the associated outcome is valuable and to refrain from responding when the associated outcome is not. Points are awarded or subtracted for correct and incorrect responses, respectively. Responses to devalued outcomes are considered slips of action and indicate an overreliance on habit. Participants were instructed that they were completing a four‐part memory task, in which they would first be learning which key press is correct for each fruit and that additional parts of the task would build on that memorization. Each phase was briefly described, and participants were told their goal was to accumulate as many points as possible throughout the task.

SOAT performance was assessed in two ways: (1) an overall total score on the task, reflecting performance on each type of trial in the task, wherein a contraindicated button press loses a point, a correct press gains a point, correct withholding to a devalued fruit gains a point or causes no change in score if the fruit is not devalued; and (2) a congruency score, reflecting the percentage of valued trials subtracted from the devalued trials (the percent inhibited slips of action minus slips of actions, wherein higher scores reflect more effective goal‐directed responses and less habit formation (de Wit et al. 2012)). However, because the two scores were highly intercorrelated (*r* = 0.99, *p* < 0.001) and results were similar across scores, we report only the total score results.

#### 2.2.3 Timeline Follow‐Back of Alcohol Use (Sobell and Sobell 1992)

Participants were asked how many drinks they had on any drinking occasion over the past 35 days (i.e., 5 weeks) in order to characterize recent drinking and ensure coverage of a full month, and four weekends, for each participant, as used in previous work (Halcomb et al. 2022; Holzhauer et al. 2022; Krenek et al. 2016; Oberlin et al. 2021; Plawecki et al. 2022).

#### 2.2.4 The Alcohol Use Disorders Identification Test (AUDIT; Saunders et al. 1993)

The AUDIT is a 10‐item self‐report screening for AUD risk, with a sum cutoff ≥ 8 reflecting clinically significant AUD risk.

#### 2.2.5 The Semi‐Structured Assessment of the Genetics of Alcoholism (Bucholz et al. 1994)

The alcohol use module was used to estimate the number of DSM‐5 AUD criteria met.

### 2.3 Procedures

#### 2.3.1 Behavioural Procedures

The study was approved by the Indiana University Institutional Review Board. Participants provided written informed consent prior to any study procedures; this study conforms with the Code of Ethics of the World Medical Association. After completing and passing a telephone screening, participants completed three testing sessions: an interview/screening session where they completed the above‐mentioned self‐report and interview measures designed to ensure inclusion criteria were met, and two counterbalanced experimental alcohol self‐administration sessions. For both experimental sessions, participants arrived at the clinical research centre in the morning and completed drug and pregnancy urine screens and a breath alcohol concentration (BrAC) measurement via a Draeger model 6510 m, after which a 22 ga. indwelling catheter was placed in the vein of the ante‐cubital fossa of one arm. A 550‐cal standardized breakfast was served, and phones and car keys were taken for safekeeping. The participant was then seated in a 5′ × 7′ Industrial Acoustic Corporation sound‐dampened chamber with a closed‐loop intercom system to enable communication between the participant and the lab technician without manual effort.

Intravenous (IV) alcohol self‐administration was used to characterize alcohol‐seeking in the human laboratory. IV alcohol administration's strengths are internal validity and experimental control, standardized brain exposure across and within individuals, greater safety at higher BrAC's, and greater power from lower BrAC variability (Cyders et al. 2020). The combination of IV alcohol self‐administration, which offers a high level of experimental control at a relative cost to ecological validity, with self‐reported alcohol use, which offers more ecological validity but is limited by self‐report accuracy, provides complementary information about the potential role of habitual responding in alcohol‐seeking. We designed this combination to represent two different aspects of alcohol use: average drinks per drinking day reflecting habitual alcohol use and work completed for IV alcohol in the laboratory reflecting motivation for alcohol.

The Computer‐Assisted Alcohol Infusion System (CAIS) software (Zimmermann et al. 2009, 2008) was used to compute the alcohol infusion rate, ensuring an identical incremental BrAC exposure for each reward delivered within and across participants through feedback and the included physiologically based pharmacokinetic model of alcohol distribution and elimination (O'connor et al. 2000; Ramchandani et al. 1999). Ethanol infusate was prepared by the university research pharmacy by mixing half‐normal saline with 95% ethanol to create a 6.0% (v/v) solution. Each session began with a 30‐min priming interval during which participants were taken to and held at an alcohol exposure of 60 mg/dL. Participants then completed a 2.5‐h alcohol self‐administration. After an initial selection of an alcohol reward for the first work set to recompute the ongoing infusion rate profile, each subsequent work set was initiated by choosing their next desired reward. Throughout each work set, participants were free to wait, work ad‐lib, pause, or cease working. Once a work set was complete, the chosen reward was administered over a 2.5‐min period. Alcohol rewards raised the participant's BrAC by 10.0 mg/dL over 2.5 min before declining at a steady rate of −0.8 mg/dL/min until the next alcohol reward was delivered or as long as was pharmacokinetically possible. Water rewards consisted of a standardized infusion of 30 mL of saline. To earn a reward, participants had to correctly respond to a pre‐set number of trials on a cognitive button‐pressing task (the Constant Attention Task [CAT]) (Plawecki et al. 2013). The number of correct trials required for reward delivery raised exponentially for each subsequent work set, with alcohol and water on separate, though identical, reward schedules. About every 10–15 min, and only during a reward delivery, participants completed a brief computerized assay of craving and subjective response to alcohol. BrAC was assessed periodically throughout the session to adjust the infusion rates to ensure fidelity to the desired exposures. Upon completion of the self‐administration paradigm, participants were given lunch and remained in an inpatient room in the hospital until at least 6:00 pm, or until their BrAC < 20 mg/dL. Participants were then paid in cash and discharged.

In one experimental session, work for alcohol and water were both paired with neutral stimuli, while in the other session, work for water was paired with neutral stimuli while work for alcohol was paired with aversive stimuli (as part of the goals of the parent study; see Garrison et al. n.d., in press, and clinicaltrials.gov [*Study Details|Human Alcohol‐Seeking Despite Aversion|ClinicalTrials. Gov*, n.d.]). To avoid confounding the results with differential stimuli presentations, the current analyses include only the session in which water and alcohol rewards were each paired with neutral stimuli.

#### 2.3.2 Magnetic Resonance Imaging (MRI) Acquisition

Participants were instructed not to think about anything in particular during the scan, to stay awake and to fixate their gaze on white crosshairs centred on a black background. Imaging was conducted on a Siemens 3 T Prisma (Erlangen, Germany) with a 64‐channel head coil array. A high‐resolution anatomical volume 3D Magnetization Prepared RApid Gradient Echo sequence (MPRAGE; Lifetime Human Connectome Protocol parameters: 1 slab with a 50% distribution factor, 208 sagittal slices/slab, slice oversampling 23.1%, 0.8 mm slice thickness, 256 mm field‐of‐view (FoV), 93.8% FoV phase, 320 × 320 matrix, repetition/echo/inversion time TR/TE/TI = 2400/2.22/1000 ms, flip angle = 8 deg, GRAPPA acceleration = 2, 0.8 × 0.8 × 0.8 mm^3^ voxels) was used for positioning the blood oxygenation level dependent (BOLD) resting state data acquisition and for image processing. Six hundred sixteen whole‐brain resting state BOLD volumes were acquired utilizing a multi‐band (MB) echo‐planar imaging (EPI) sequence (Center for Magnetic Resonance Research at the University of Minnesota, gradient echo, TR/TE = 780/29 ms, flip angle 54 deg, field‐of‐view 220 × 220 mm^2^, matrix 88 × 88, 55 2.5 mm thick slices, 2.5 × 2.5 × 2.5 mm^3^ voxel, slice acceleration factor = 5) (Smith et al. 2013). For each participant, the first 20 volumes of resting state BOLD data were excluded from analyses to ensure that steady state magnetization state was reached. BOLD functional MRI (fMRI) acquisition was preceded by a pair of phase‐reversed spin echo field mapping scans (3 A‐P and 3 P‐A phase direction volumes, TR/TE = 1200/64.40 ms); other imaging parameters matched the BOLD fMRI acquisition.

#### 2.3.3 Image Preprocessing

Preprocessing was completed with an in‐house Bash and Python 3.6.8 based pipeline using FMRIB Software library (FSL version 6.0.1). T1‐weighted MPRAGE image of each participant was denoised prior to brain masking and extraction with Advanced Normalization Tools (ANTs) (Avants et al. 2009) and then nonlinearly transformed (FSL's *flirt* and *fnirt*) to Montreal Neurological Institute (MNI) brain template space. This MNI‐to‐T1 transformation was followed by T1‐to‐EPI transformation (see EPI preprocessing) allowing us to perform standard‐to‐native (i.e., MNI‐to‐EPI) space and inverse (EPI‐to‐MNI) space transformations required for the region of interest analysis (see next section). Resting state FC (rsFC) data were preprocessed in native BOLD EPI space of each participant, including BOLD volume distortion correction using FSL's *topup/applytopup* (utilizing phase‐reversed spin echo field mapping scans), head motion realignment (*mcflirt*), T1‐to‐EPI registration (linear, nonlinear, and boundary‐based registrations), normalization to mode 1000 and spatial smoothing with a 6‐mm isotropic full width at half maximum (FWHM) Gaussian kernel.

Following recommendations for robust preprocessing (Eklund et al. 2016), the preprocessed data were entered into FSL's MELODIC (Nickerson et al. 2017) for independent components analysis (ICA)‐based denoising with ICA‐AROMA (Pruim, Mennes, Buitelaar, and Beckmann 2015; Pruim, Mennes, van Rooij, et al. 2015). A single step regression was applied to the denoised BOLD volumes to avoid reintroducing artefacts in the preprocessed denoised data (Lindquist et al. 2019; Phạm et al. 2023). Specifically, regressors were applied that (1) indexed head motion (from the realignment and their derivatives) (Power et al. 2014), (2) accounted for physiological noise (first five signals obtained by principal components analysis from the white matter and cerebrospinal fluid‐eroded masks; an implementation of aCompCor) (Muschelli et al. 2014), (3) performed high‐pass filtering (*f*_*min*_ = 0.009 Hz) using Discrete Cosine Transforms bases (Shirer et al. 2015) and (4) included outlier volume despiking (Phạm et al. 2023). The outlier despiking was based on the significant ‘DVARS’ metrics obtained on the single‐regression preprocessed data, which tagged a mean of 1.17% (SD = 0.85) of residual high head motion volumes; these outlier volumes were excluded from the calculation of correlation coefficients.

#### 2.3.4 Regions of Interest

**FIGURE 1:**
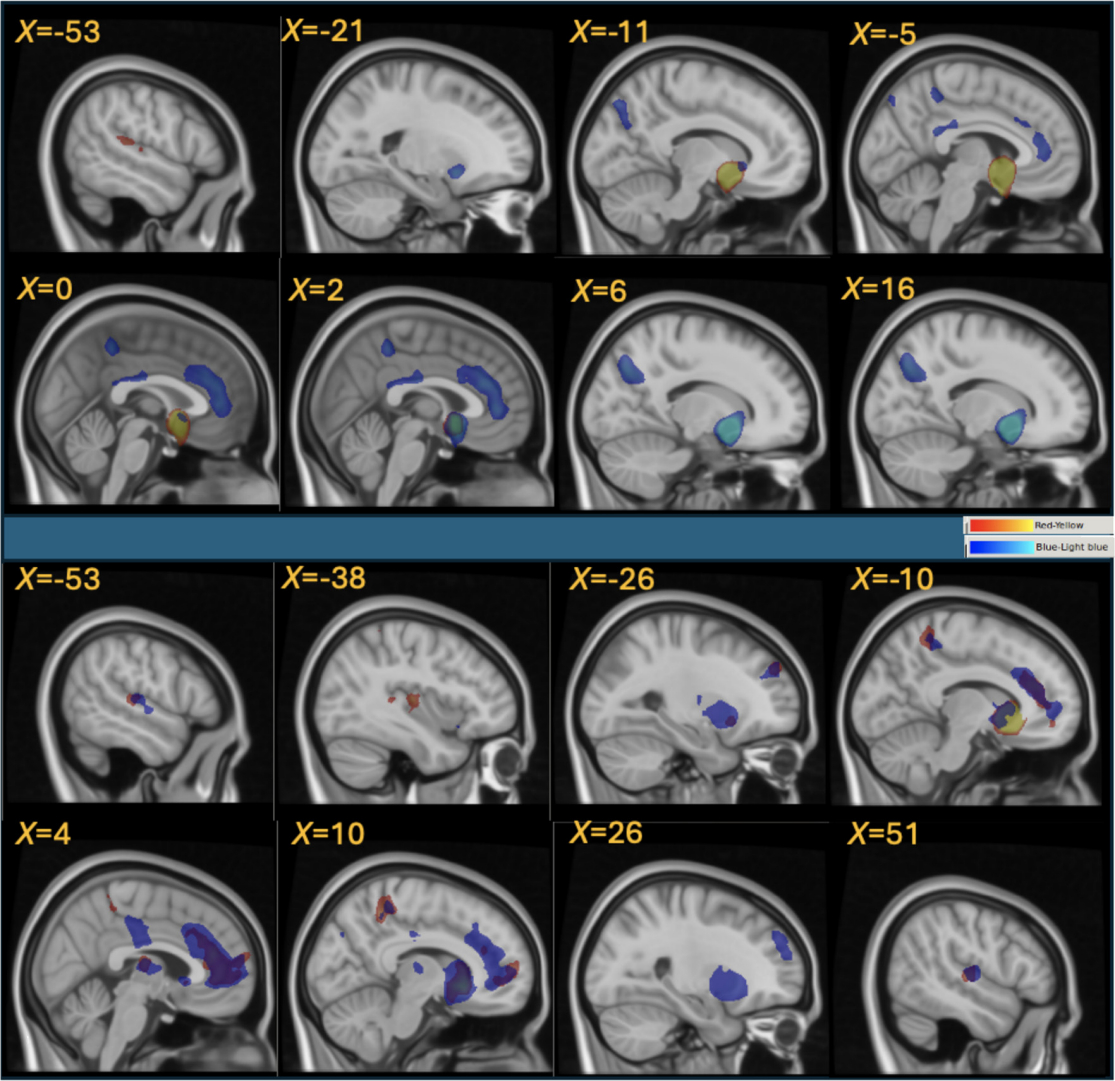
Functional connectivity for nucleus accumbens and ventral caudate seeds. Functional connectivity (FC) of the left and right nucleus accumbens (L and R NAc) is illustrated in the top eight panels. Left NAc shows significant FC to the left parietal operculum (yellow‐red). Right NAc exhibits prominent FC to the contralateral ventral putamen, bilateral precuneus, medial postcentral gyrus and posterior and anterior cingulate cortex (blue‐light blue). The ventral caudate (vCau) seeds (bottom eight panels) show largely symmetric and overlapping FC pattern, with the most prominent FC to the anterior cingulate cortex. For details, see Table S1. Displayed at voxel‐level *p*_*FWE*_ < 0.05, *k* = 250. FWE = family‐wise error corrected. k = minimum cluster size (in mm^3^). *t*‐Statistic range in the colour bars is 5.34–8.

We used three bilateral medial striatal seeds in the NAc, dorsal caudate and ventral caudate (see Figure S1). The seed regions were defined using the Melbourne subcortical atlas, based on functionally connectivity fingerprinting of the resting state data from 1080 healthy control participants (Tian et al. 2020). We chose this parcellation because it has been demonstrated both with functional MRI and previous immunohistochemical studies (Cartmell et al. 2019; Meredith et al. 1996) that human functional and structural distinctions exist between these regions. The differences in the connectivity patterns of these seed regions follow seminal work by Haber (2016), Haber et al. (2006) and a recent study by Peng et al. (2024) who used tract‐tracing data from non‐human primates to assess their representation in resting state fMRI data from non‐human primates and humans. Our NAc and caudate seeds are broadly consistent with these studies and show robust connectivity to the contralateral striatal regions, cingulate cortex, frontoinsular and cerebellar (dorsal caudate seed only) areas as illustrated by Figure 1. Prior to hypothesis testing, we verified FC patterns of each seed with the meta‐analytic coactivation map, which reflects coactivation of brain regions across studies in the Neurosynth database (Yarkoni et al. 2011). We centred 6 mm radius spheres on the seed centroid locations and obtained z‐score maps showing voxels most likely to be activated in the same studies as the centroid voxel.

Seed regions (in 1 mm standard MNI space) were nonlinearly transformed to native EPI space as described in the previous section. Mean BOLD time series from each seed were extracted and correlated with all brain voxels' time series in native space to generate whole‐brain Pearson's correlation coefficient maps. A *z*‐statistic map was then calculated with a variance‐stabilizing Fisher *z*‐transformation and smoothed by a 3 mm isotropic FWHM Gaussian kernel, which together with the earlier described pre‐AROMA smoothing resulted in the overall 6.7 mm isotropic FWHM Gaussian kernel smoothing. Finally, native space *z*‐statistic maps of each participant were transformed to MNI space using previously described FSL registrations.

#### 2.3.5 Data Analysis Approach

After data were checked for accuracy, all subscales on the above‐mentioned self‐report scales were calculated and variable distributions (e.g., skewness, kurtosis, outliers) and sample characteristics were examined. Work for alcohol was calculated as the total number of CAT trials completed for alcohol during the alcohol self‐administration session. To test Hypothesis 1, bivariate correlations were calculated among the SOAT score, cumulative work for alcohol in the laboratory session and average number of drinks per drinking day. To test Hypothesis 2, FSL‐derived *z*‐statistic images for each participant and seed were entered in voxel‐wise factorial model in Statistical Parametric Mapping (SPM) software version 12, with the seed hemisphere as a factor (2 levels: Left, Right, specified to be dependent), the SOAT score as a regressor interacting with the hemisphere and age and biological sex as covariates. FC and whole‐brain FC associations of the seed regions with SOAT score were assessed using a family‐wise error (FWE) corrected cluster‐level *p*_*FWE*_ < 0.05 significance at a cluster‐forming threshold *p* = 0.001 and using a Bonferroni correction of *p* < 0.008. To test Hypothesis 3, two parallel mediation analyses were conducted in Statistical Product and Service Solutions (SPSS) software using the PROCESS macro, with SOAT score as the independent variable, alcohol use (average drinks per drinking day and cumulative work for alcohol tested in separate models) as the dependent variable, and means of the significant FC clusters as the mediators (entered simultaneously). Indirect effects with 95% confidence intervals not crossing zero were deemed significant.

## 3 Results

### 3.1 Study Variables and Bivariate Correlations

Skewness and kurtosis were within normal limits for all study variables. Participants reported an average of 4.60 (SD = 1.89) drinks per drinking occasion in the previous 35 days and completed a mean of 249.69 (SD = 222.72) trials for alcohol rewards cumulatively across the 2.5‐h alcohol session. On average, participants endorsed hazardous alcohol use (mean AUDIT = 10.36, SD = 5.25) and 55.6% of our sample met DSM‐5 criteria for AUD, with participants, on average, endorsing 2.25 criteria (SD = 2.42). SOAT score was not significantly correlated with average drinks per drinking day (*r* = −0.28, *p* = 0.10) or with cumulative work for alcohol rewards (*r* = −0.12, *p* = 0.50).

### 3.2 FC of Seed ROIs and Lateralization (Tables S1 & S2, Figure 1)

Significant FC clusters for each seed in this study are listed in Tables S1 and S2. Our FC results are consistent with the Neurosynth meta‐analysis co‐activations, with ‘connectivity hotspots’ in the anterior cingulate/medial prefrontal cortex, precuneus and parietal operculum/posterior insula as illustrated by Figure 1 and Figure S1. To best illustrate these connectivity findings (Figure 1, Figure S2) and present only the largest, most prominent FC foci listed in Supplemental Tables, we used voxel‐wise whole‐brain corrected significance, *p*_*FWE*_ < 0.05, and threshold of *k* = 250 for our visualization and the Supplemental Tables. When FC for the left and right seeds was directly compared to each other, there were no significant hemispheric differences in connectivity for any of the seeds. This hemispheric symmetry in FC persisted at less stringent *p*_uncorr_ < 0.001 (data not shown).

### 3.3 Associations Between FC and SOAT (Table 1)

**TABLE 1:** Associations between Slips of Action Task and functional connectivity strength of seed regions.

Seed	Condition	Region	Cluster		Peak	Peak MNI coordinate		
			*p (FWE)*	*k*	*Z*	x (mm)	*y (mm)*	*z (mm)*
L NAc	SOAT(+)	R CO	**0.005**	1892	4.05	54	−7	12
		R CO			3.82	62	−4	5
		R STG			3.81	65	4	0
		L CO	0.043	1190	3.90	−54	−10	8
		L PoG	0.019	1437	3.84	−67	−18	31
		L PoG			3.72	−65	−22	15
		L PoG			3.43	−60	−9	43
L vCau	SOAT(−)	L MFG	**0.002**	2174	4.50	−31	0	55
		L PrG			4.14	−29	−13	52
		L SFG			3.91	−24	8	63

*Note:* Significance was assessed using cluster‐level *p*_*FWE*_ < 0.05 at a cluster‐forming threshold *p* < 0.001 (uncorrected). (+) indicates a positive association and (−) indicates a negative association. No significant associations were observed for the right seed regions. The bolded values are the *p* *_FWE_* 〈 0.05 clusters.

Abbreviations: CO = central operculum; FWE = family‐wise error correction for multiple comparisons, *k* = 250; minimum cluster size (mm^3^); L = left; MFG = middle frontal gyrus; MNI = Montreal Neurological Institute; NAc = nucleus accumbens; PoG = postcentral gyrus; PrG = precentral gyrus; R = right; SFG = superior frontal gyrus; SOAT = Slips of Action Task total score; STG = superior temporal gyrus; vCau = ventral Caudate.

**FIGURE 2:**
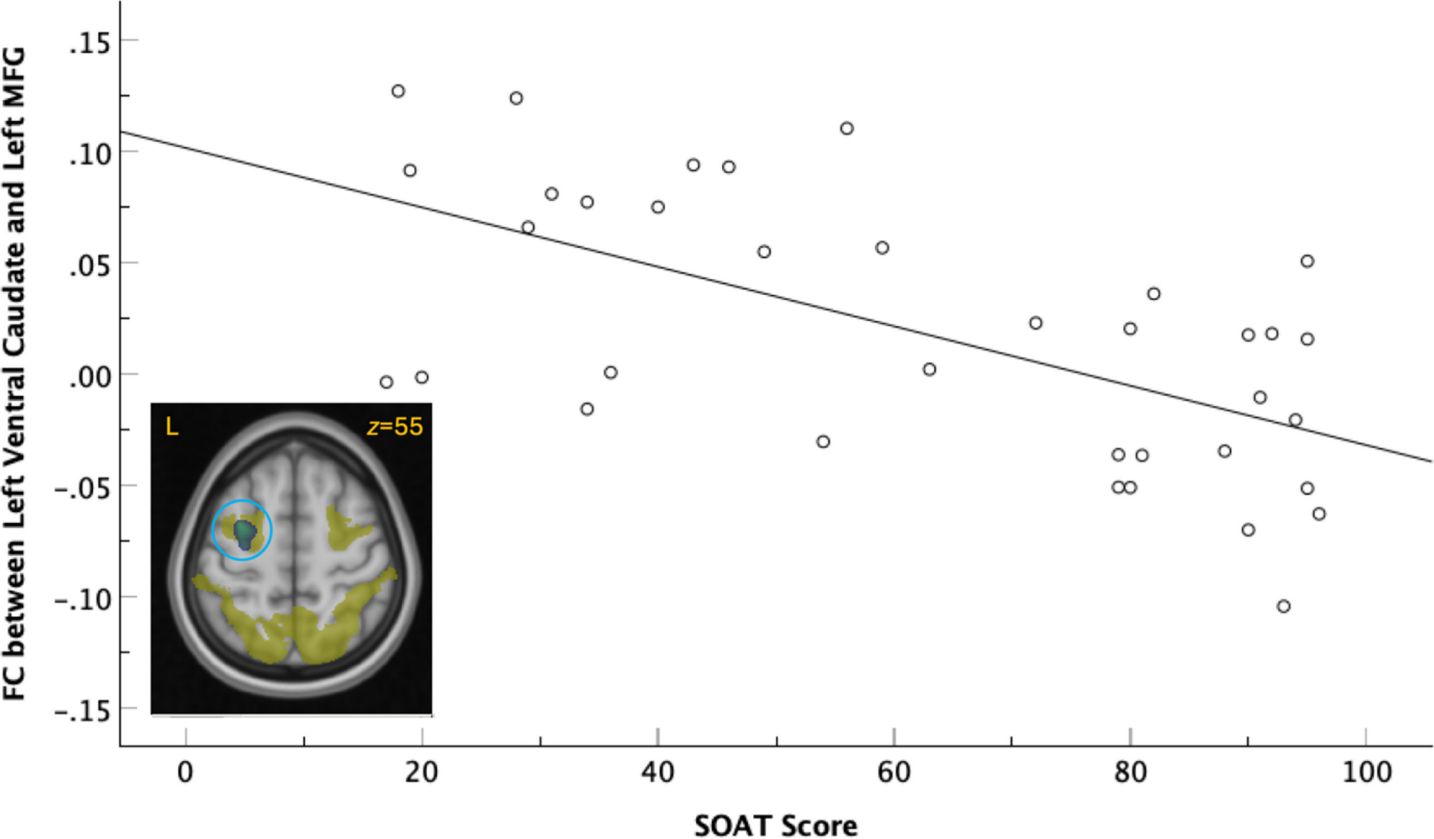
Functional connectivity (FC) strength between the left ventral caudate seed and the left middle frontal gyrus (MFG) is negatively associated with the total Slips of Action Task (SOAT) score. The cluster, indicated by a light blue circle, has the primary peak in the MFG ([−31, 0, 55]) that extends into the left superior frontal gyrus (peak at [−24, 0, 63]). Overlap (green) of the cluster (blue‐light blue) and dorsal attention network (yellow). L = left. Fit line represents bivariate correlation and is shown for illustration purposes only.

FC strength between the left ventral caudate seed and the left prefrontal region of the middle frontal gyrus (MFG), which extended into the precentral gyrus, showed a significant cluster of negative association with SOAT score (cluster *p*_*FWE*_ = 0.002, *k* = 2174) that survived Bonferroni correction (cluster *p*_*FWE*_ = 0.008; Figure 2). This relationship was left‐dominant; the FC strength between the right ventral caudate seed and left MFG was not significantly associated with SOAT score (cluster *p*_*FWE*_ = 0.175, *k* = 786). Testing (left > right) lateralization of the left MFG cluster association with the SOAT score did not yield corrected cluster significance (*p*_*FWE*_ = 0.18) but resulted only in an uncorrected cluster significance (*p*_*uncorr*_ = 0.009).

**FIGURE 3:**
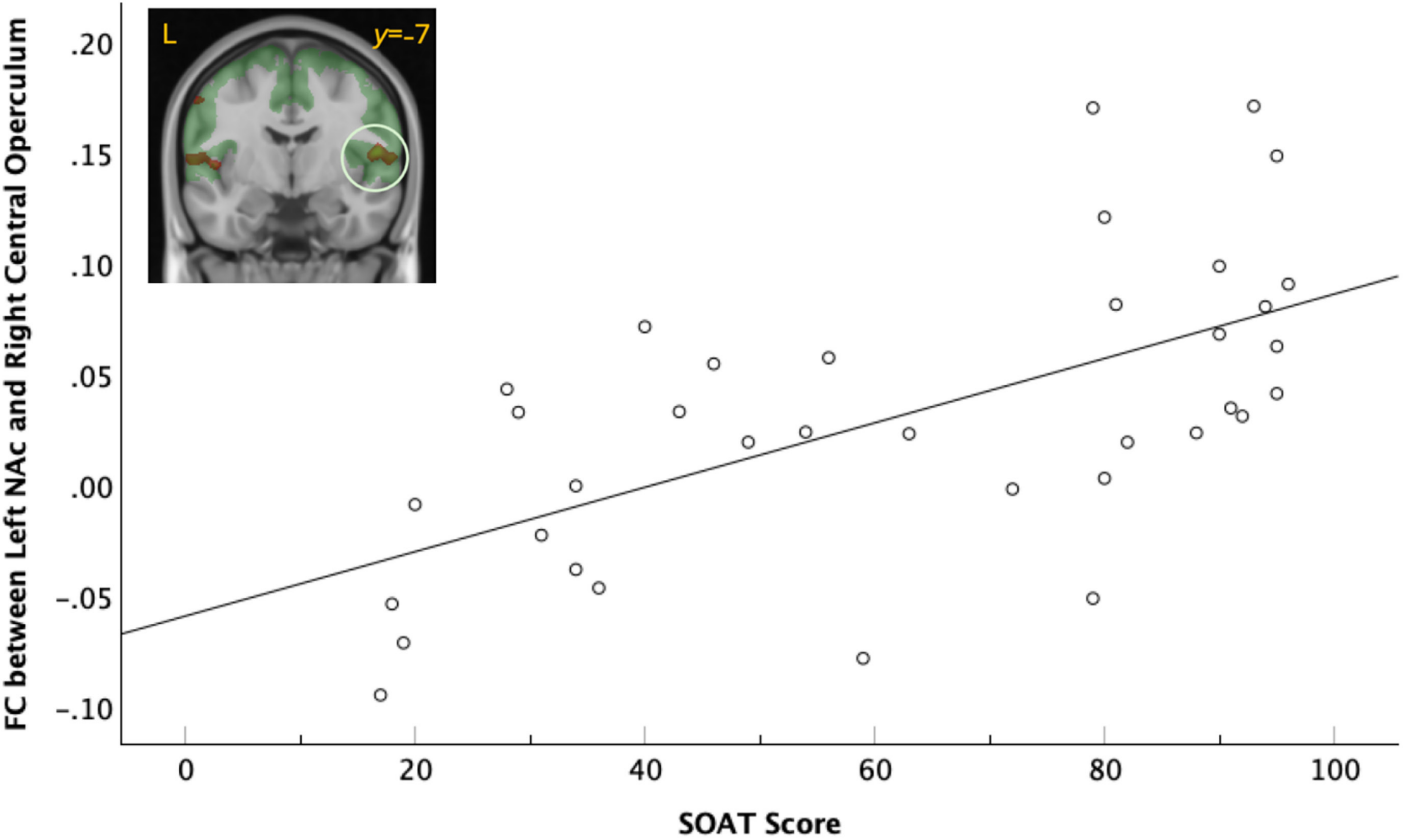
Functional connectivity (FC) strength between the left nucleus accumbens (NAc) seed and the right central operculum (CO, light green circle) is positively associated with the total Slips of Action Task (SOAT) score. Overlap of the SOA cluster (red‐yellow) and somatomotor network (green). L = left. Fit line represents bivariate correlation and is shown for illustration purposes only.

FC between the left NAc seed and the right central operculum (CO) showed a significant cluster of positive association with SOAT score (cluster *p*_*FWE*_ = 0.005, *k* = 1892) that survived Bonferroni correction (cluster *p*_*FWE*_ = 0.020) (Figure 3). Again, this relationship was left‐dominant; the FC strength between the right NAc seed and right CO was not significantly associated with SOAT score (cluster *p*_*FWE*_ = 0.856, *k* = 287). Testing (left > right) lateralization of the R CO cluster association with SOAT score did not reach significance (corrected cluster significance *p*_*FWE*_ = 0.86, *p*_*uncorr*_ = 0.091).

FC of the right ventral caudate, right NAc and the right and left dorsal caudate seeds did not show any significant clusters correlated with the SOAT score (see Tables S1 and S2).

### 3.4 Parallel Mediation Models

**FIGURE 4:**
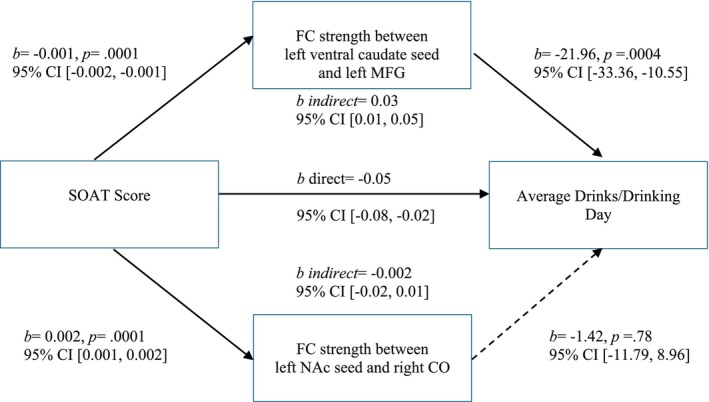
Parallel mediation model on average drinks per drinking day. CO = central operculum; MFG = middle frontal gyrus; NAc = nucleus accumbens; SOAT = Slips of Action Task. Solid arrow indicates a significant association. Dashed arrow indicates nonsignificant association.

The two significant clusters (left ventral caudate and left MFG, left NAc and right CO) were entered simultaneously into a parallel mediation analysis. The relationship between SOAT score and average drinks per drinking occasion was significantly mediated by the FC strength between the left ventral caudate seed and the left MFG (indirect *b* = 0.03 [95% *CI* 0.01, 0.05]; see Figure 4). The FC strength between the left NAc seed and the right CO was significantly related to SOAT score but was not a significant mediator, as it was not significantly related to average drinks per drinking day (*b* = −1.42, *p* = 0.78).

**FIGURE 5:**
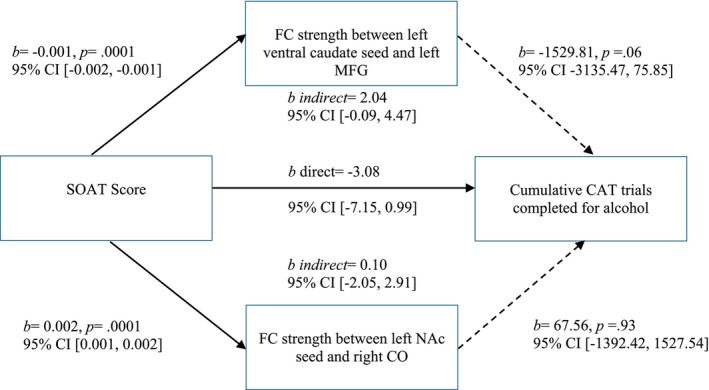
Parallel mediation model on cumulative work for alcohol. CAT = constant attention task; CO = central operculum; MFG = middle frontal gyrus; NAc = nucleus accumbens; SOAT = Slips of Action Task. Solid arrow indicates a significant association. Dashed arrow indicates nonsignificant association.

Neither seed significantly mediated the relationship between SOAT score and cumulative work for alcohol. However, FC between the left ventral caudate and left MFG was significantly related to SOAT score (*b* = −0.001, *p* = 0.0001), and fell just short of significance with cumulative work for alcohol (*b* = −1592.81, *p* = 0.06) and as a mediator (indirect *b* = 2.04 [95% *CI* ‐0.09, 4.47]) (see Figure 5). FC between the left NAc and the right CO was not a significant mediator, as it was not significantly related to cumulative work for alcohol (*b* = 67.56, *p* = 0.93).

## 4 Discussion

This study provides evidence that the SOAT is significantly related to FC of left ventral caudate among individuals who use alcohol heavily, and that this connectivity patterns in part explains the relationship between habitual responding and recent drinking. This study extends previous structural and functional work with the SOA task to a resting state approach, which can provide a more comprehensive understanding of the brain's baseline (intrinsic) network connectivity and organization. Although it fell short of significance, there was evidence that this underlying FC may also explain the relationship between habitual responding and alcohol‐seeking in the human laboratory, which may catalyse future research. Implementing this work in the human alcohol laboratory is an important extension of this area of research.

Performance on the SOAT was significantly related to the resting state FC strength between left ventral caudate and the left MFG, such that better performance on the SOAT (i.e., more goal‐directed and less habitual behaviour) was related to *less* FC between the left ventral caudate and left prefrontal MFG area. The ventral caudate is involved in reward processing and goal‐directed behaviours, specifically in decision‐making and motivation during reward‐related learning (Castro and Bruchas 2019). The MFG is involved in attention (Japee et al. 2015; Thiel et al. 2004) and our result was in the dorsal attention resting state network (Yeo et al. 2011). There is some evidence that the left MFG may reflect inhibitory errors and prospectively predict early problematic substance use (Heitzeg et al. 2014), playing a key role in goal‐directed behaviours (Grahn et al. 2008). Functional coupling of the caudate and dorsal frontal cortex has been linked with responses to smoking cues, suggesting that top–down modulation of reward processing may underlie craving (Yuan et al. 2017), a general diminished attentional control, inefficient integration of reward and action, or over‐reliance on reward processing. de Wit et al. (2012) found that white matter tracts between the caudate and the ventromedial prefrontal cortex were linked to *more goal‐directed* behaviour using the SOAT, suggesting integration across caudate and frontal regions in the reinforcement of action taken toward rewards (Grahn et al. 2008). However, in this study, resting state synchronicity between the caudate and the MFG is linked with *more habitual* behaviour and *less alcohol use and seeking*. Differences between these findings may be due to a number of factors, including different connectivity metrics (structural vs. functional), the use of a ventral subdivision of the caudate in the current analysis and differential functional role of the reported frontal regions (ventromedial prefrontal cortex vs. MFG). The resting state connectivity found here may also reflect the sample of study, as altered resting state synchronicity is found in many clinical disorders, including AUD and may reflect brain neuroadaptation that underlie psychopathology (Whitfield‐Gabrieli and Ford 2012).

We also found that SOAT was significantly related to the resting state FC strength between the left NAc and the right CO, such that better performance on the SOAT (i.e., more goal‐directed and less habitual behaviour) was related to *more* FC between the left NAc and central opercular cortex, but did not significantly relate to alcohol use outcomes. The NAc is heavily involved in reward processing, anticipation and motivation, while the CO is involved in sensory processing, interoception and emotional regulation, and is part of the resting state somatomotor network (Yeo et al. 2011). Stimulation of the right frontal operculum elicits somatosensory and auditory symptoms (Mălîia et al. 2018), signals likely to be integrated and processed within the larger cingulo‐opercular (salience) network to which this cortical region belongs (Uddin et al. 2019). This positive relationship means that more goal‐directed behaviour was linked with a more resting state synchronized brain network that integrates reward motivation and integration of sensory and motor information, supporting effective pursuit and regulation of goals.

Previous evidence linking the SOAT to impulse control‐related clinical disorders and behaviours (Zhukovsky et al. 2020) primarily utilized self‐reported drug and alcohol use or comparisons between those who demonstrate risk‐taking behaviours as compared to healthy controls. This study extends the previous literature by using laboratory‐based progressive ratio responding; however, we failed to find a relationship with alcohol‐seeking, and the mediation by FC between the left ventral caudate and the left MFG fell short of significance. This contradicts a wealth of literature linking habitual responding with alcohol self‐administration across the preclinical literature (Corbit et al. 2012; Dickinson et al. 2002; Mangieri et al. 2012). Habitual behaviour is commonly modelled preclinically with free choice drinking paradigms (Corbit et al. 2012; Cuzon Carlson et al. 2011; Dickinson et al. 2002; Mangieri et al. 2012); our alcohol‐seeking paradigm used a progressive‐ratio design, which likely reflects motivated, goal‐directed alcohol‐seeking (Cyders et al. 2021), which may explain our null results. Examining SOAT with a free access alcohol task may better match habit and may provide better translation with preclinical work (Cyders et al. 2021). Several study design factors may have further contributed to lack of a relationship, including the use of an alcohol prime of 60 mg/dL, which may have influenced the motivation in the self‐administration portion of the study, and limiting our sample to individuals who drink alcohol heavily, which could have reduced power to detect effects.

Although the usefulness of the SOAT for human alcohol‐seeking laboratory paradigms was not strongly supported, additional work, including in oral alcohol challenge and alternative IV alcohol‐based approaches (e.g., free access, rate control; see (Cyders et al. 2020)), and with alcohol‐specific stimuli (Sjoerds et al. 2013) should be conducted before making a final conclusion on the applicability of the SOAT for human alcohol laboratory‐based studies. Further testing is key, as the SOAT has the potential to facilitate translation between preclinical and clinical research (as suggested by (de Wit and Dickinson 2009; Halcomb et al. 2019)), the current sample was modest and of limited power, and the progressive ratio task may have reflected goal‐directed, rather than habitual, alcohol‐seeking. Insofar as habitual responding reflects compulsive behaviours (Van Timmeren et al. 2018), the SOAT may serve as an objective behavioural marker that is not conflated with alcohol‐seeking and that could be used to complement self‐reported compulsive behaviours. Another study examined a real‐life extension of the SOAT that showed promising results linking the SOAT to real‐life slips of action that could additionally inform multi‐modal and multi‐setting research (Linnebank et al. 2018).

Our findings did not replicate previous work with the SOAT that showed correspondence with dorsal caudate responses (de Wit et al. 2012; Watson et al. 2018; Zhukovsky et al. 2020).

Previous studies examining neuroimaging correlates of the SOAT have used task‐based designs, where participants were imaged during the completion of the SOAT. Such designs offer the advantage of understanding the extent to which specific brain regions are activated during a specific task (e.g., SOAT). Resting‐state designs, as used in this work, are less specific but offer a broader understanding of the brain's baseline state. They provide insights into how different brain regions naturally coordinate into resting‐state networks (Yeo et al. 2011) that regulate cognitive and emotional processes, including brain activity that task‐based paradigms do not capture. In addition, resting state scans are generally easier to conduct and compare across various studies allowing for the inclusion of larger and more diverse populations (e.g., Human Connectome Project, UK Biobank, ABCD study). In our study, the resting‐state data were complemented by SOAT data acquired in a separate session and outside the scanner, allowing more accurate behavioural assessment in a controlled laboratory environment and with lower time and cost. In summary, we view the combination of task‐based and resting state fMRI designs as complementary, providing richer and more comprehensive understanding of brain function.

Although our FC results showed no hemispheric asymmetry for any of the seed regions, the associations between FC and SOAT were observed only in the left hemisphere seeds. Korponay et al. (2021) previously documented greater leftward connectional laterality in the right rostral ventral putamen, left rostral central caudate and bilateral caudal ventral caudate, and that, across participants, greater leftward connectional laterality at the left rostral caudate hotspot was associated with higher performance on tasks engaging lateralized functions (i.e., response inhibition and language, respectively). Previous work has highlighted response inhibition, often measured via a stop signal or go/no‐go task, as a right‐lateralized process (Aron et al. 2004, 2014; Garavan et al. 1999; Ocklenburg et al. 2011). However, emerging work has highlighted that response inhibition is not a unitary construct, comprising separate motor, cognitive and emotion sets that may be governed by different frontal regions and may be differentially lateralized, with more cognitive domains being left‐lateralized and more emotional domains being right‐lateralized (Dillon and Pizzagalli 2007; Shallice and Cipolotti 2018). Similarly, one recent study found that inhibition (i.e., suppressing a response) and a related construct known as shifting (i.e., shifting between different cognitive tasks) are differentially lateralized in the right and left hemispheres, respectively (Rodríguez‐Nieto et al. 2022). Thus, the SOAT, which is cognitive in nature and requires adjusting rules and behaviour, may result in left, rather than right, lateralized FC associations. Given work with the SOAT is emerging, this should be studied further.

These findings should be understood within study limitations, including the sample size, which limits power to detect effects, but still provides good estimates for future research planning. In addition, the cross‐sectional nature of the study precludes establishing causal relationships. Participants endorsed moderate‐to‐heavy drinking, so these results may not generalize to light drinkers, those who are alcohol naïve, or those with AUD. Due to the lack of a reference group, we also cannot conclude that the findings are specific to heavy drinkers alone. Although temporal precedence for the mediation models was selected based on previous theory, the relationships supported by the current study should be tested in future prospective models. Resting state analyses examine the brain while not engaging in a designated task or activity, which may contribute to variation in the extent to which the participant was truly ‘at rest’ during the scan and cannot answer the question of how the brain might respond while engaging in the SOAT (which has previously been studied by Watson et al. (2018)). Future work should incorporate structural, task‐based and resting state imaging in the same participants in order to better understand between‐study differences.

In conclusion, habit formation's influence on alcohol use may function in part through neuroadaptations in the left ventral caudate. More work is needed to better characterize the SOAT with additional laboratory‐, alcohol‐specific, imaging‐ and ambulatory‐based alcohol use metrics to establish whether the SOAT has the potential to facilitate translation between preclinical and clinical AUD research.

## Author Contributions

**Lindsey R. Fisher‐Fox:** formal analysis, writing – original draft, writing – review and editing. **Mario Dzemidzic:** conceptualization, formal analysis, funding acquisition, investigation, methodology, project administration, software, supervision, writing – review and editing. **McKenzie R. Cox:** project administration, writing – review and editing. **David Haines:** project administration. **James Hays:** project administration, writing – review and editing. **Mayande K. Mlungwana:** project administration. **Zachary Whitt:** project administration. **Andrea Avena‐Koenigsberger:** software. **Ann E. K. Kosobud:** funding acquisition, project administration, writing – review and editing. **David A. Kareken:** conceptualization, funding acquisition, supervision, writing – review and editing. **Sean O'Connor:** conceptualization, funding acquisition, writing – review and editing. **Martin H. Plawecki:** conceptualization, funding acquisition, project administration, supervision, writing – review and editing.

## Conflicts of Interest

The authors declare no conflicts of interest.

### Peer Review

The peer review history for this article is available at https://www.webofscience.com/api/gateway/wos/peer‐review/10.1111/ejn.70150.

## Supporting information


**Table S1.** Functional connectivity of the NAc and ventral caudate (vCau) seeds.
**Table S2.** Functional connectivity of the dorsal caudate seeds.
**Figure S1.** Functional connectivity (FC) of the nucleus accumbens and caudate seeds. Nucleus accumbens (NAc; red), ventral caudate (vCau; yellow) and dorsal caudate (dCAu; green) seed regions were defined using the Melbourne Scale II subcortical atlas. The right and left region labels are: NAc (9, 25), vCAu (10, 26) and dCAu (15, 31). The MNI coordinates indicate location of brain slices and the crosshairs. Presented in neurological orientation.
**Figure S2.** Functional connectivity (FC) of the dorsal caudate (dCau) seeds; (left: red‐yellow; right: blue‐light blue). Both dCau seeds show prominent connectivity to the contralateral striatum and medial (−12, −6, and 6 sagittal views) premotor and anterior cingulate/prefrontal areas, as well as the cerebellum. The left dCau FC foci are in the left inferior frontal, insula and frontopolar areas. The right dCau seed also show FC to the insula (x = 40) and right orbital areas. Illustrated at voxel‐wise *p*_*FWE*_ < 0.05, *k* = 250). *t*‐Statistic range in the colour bars is 5.34–8. Also see Table S2.

## Data Availability

The data that support the findings of this study are available from the corresponding author upon reasonable request.
