# Supplementary material for: Left Ventral Caudate Functional Connectivity Mediates the Relationship Between Habitual Responding and Alcohol Use
Source: Eur J Neurosci. 2025 May 26;61(10):e70150. doi: 10.1111/ejn.70150 (PMC12104795; doi:10.1111/ejn.70150)

Supplemental Table 1. Functional Connectivity of the NAc and Ventral Caudate (vCau) Seeds.

| Seed | Region | Cluster | Peak | | Peak MNI coordinate | | |
| --- | --- | --- | --- | --- | --- | --- | --- |
|  |  | *k* | *p(FWE)* | *Z* | *x(mm)* | *y(mm)* | *z(mm)* |
| **L NAc** | L NAc | 8189 | <0.001 | >8.00 | -6 | 8 | -8 |
| Centroid:  [-10,10,-6] | L vCau |  | 0.030 | 4.98 | -14 | 23 | -6 |
|  | L PT/PO | 589 | 0.001 | 5.65 | -53 | -31 | 13 |
|  | L PT |  | 0.009 | 5.24 | -56 | -23 | 9 |
| **R NAc** | R NAc | 11124 | <0.001 | >8.00 | 10 | 9 | -9 |
| Centroid: [12,10,-6] | R Amg |  | 0.005 | 5.36 | 26 | 0 | -10 |
|  | R Put |  | 0.009 | 5.25 | 23 | 18 | 3 |
|  | L Put | 1344 | <0.001 | 6.52 | -21 | 11 | -10 |
|  | L vCau |  | 0.001 | 5.81 | -11 | 19 | -2 |
|  | R PCu | 3990 | <0.001 | 6.44 | 16 | -69 | 35 |
|  | R PCu |  | <0.001 | 6.11 | 20 | -59 | 30 |
|  | R PCu | 2331 | <0.001 | 6.38 | 6 | -42 | 53 |
|  | R PCu |  | 0.002 | 5.61 | 8 | -48 | 45 |
|  | R ACgG | 8288 | <0.001 | 6.37 | 3 | 33 | 23 |
|  | R ACgG |  | <0.001 | 6.34 | 3 | 43 | 3 |
|  | L ACgG |  | <0.001 | 6.22 | -2 | 42 | 10 |
|  | L PCgG | 2087 | <0.001 | 5.84 | -6 | -43 | 24 |
|  | L PCgG |  | 0.001 | 5.78 | -7 | -30 | 30 |
|  | R PCgG |  | 0.001 | 5.76 | 5 | -42 | 22 |
|  | L SOC | 1542 | <0.001 | 5.77 | -11 | -78 | 45 |
|  | L PCu |  | 0.005 | 5.39 | -11 | -74 | 37 |
|  | L PCu |  | 0.010 | 5.22 | -15 | -72 | 30 |
| **L vCau** | L vCau | 9334 | <0.001 | >8.00 | -12 | 18 | -5 |
| Centroid:  [-12,18,-6] | L ACgG |  | 0.002 | 5.51 | -11 | 30 | -4 |
|  | L Amg |  | 0.006 | 5.34 | -17 | -1 | -12 |
|  | R PCu | 4919 | <0.001 | 7.32 | 12 | -46 | 50 |
|  | R PoG/PCu |  | <0.001 | 6.37 | 10 | -40 | 56 |
|  | L PCu/PoG |  | <0.001 | 6.22 | -4 | -46 | 67 |
|  | R NAc | 5605 | <0.001 | 7.15 | 14 | 18 | -6 |
|  | R dCau |  | <0.001 | 6.71 | 13 | 18 | 3 |
|  | R dCau |  | 0.001 | 5.77 | 14 | 14 | 16 |
|  | R Tha | 933 | <0.001 | 6.98 | 2 | -14 | 12 |
|  | L ACgG | 16791 | <0.001 | 6.81 | -6 | 36 | 20 |
|  | R ACgG |  | <0.001 | 6.46 | 2 | 40 | 20 |
|  | FP |  | <0.001 | 6.46 | 0 | 66 | 17 |
|  | L pIns | 2801 | <0.001 | 6.27 | -38 | -18 | 8 |
|  | L PO |  | 0.001 | 5.69 | -45 | -31 | 15 |
|  | L PT |  | 0.001 | 5.63 | -52 | -26 | 7 |
|  | R HG/PT | 1386 | <0.001 | 6.10 | 51 | -19 | 4 |
|  | R HG/PT |  | 0.008 | 5.26 | 42 | -24 | 4 |
|  | L FP | 479 | <0.001 | 5.86 | -26 | 50 | 33 |
|  | R vIns | 275 | 0.001 | 5.61 | 34 | 20 | -11 |
|  | PrG/PCgG | 407 | 0.002 | 5.58 | 0 | -16 | 47 |
|  | L SPL | 285 | 0.003 | 5.45 | -35 | -46 | 42 |
| **R vCau** | R vCau | 18900 | <0.001 | >8.00 | 15 | 18 | -5 |
| Centroid: [14,18,-6] | R FO/aIns |  | <0.001 | 6.78 | 31 | 22 | -10 |
|  | R Put |  | <0.001 | 6.57 | 30 | -1 | -6 |
|  | L Put | 10800 | <0.001 | >8.00 | -22 | 11 | -9 |
|  | L NAc |  | <0.001 | 6.40 | -9 | 8 | -5 |
|  | L GP |  | <0.001 | 6.00 | -22 | -10 | 5 |
|  | R ACgG | 29390 | <0.001 | 7.48 | 3 | 39 | 22 |
|  | R ACgG |  | <0.001 | 7.36 | 4 | 32 | 31 |
|  | L ACgG |  | <0.001 | 7.06 | -4 | 36 | 19 |
|  | R Tha | 1480 | <0.001 | 6.67 | 2 | -13 | 12 |
|  | R Tha |  | <0.001 | 6.06 | 2 | -5 | 8 |
|  | R PCgG | 3120 | <0.001 | 6.39 | 7 | -13 | 31 |
|  | R PCgG |  | <0.001 | 6.31 | 8 | -22 | 35 |
|  | R PCgG |  | <0.001 | 5.99 | 4 | -24 | 44 |
|  | R HG | 1128 | <0.001 | 6.20 | 52 | -15 | 3 |
|  | R PoG | 527 | <0.001 | 5.97 | 10 | -39 | 57 |
|  | R PCu |  | 0.012 | 5.19 | 11 | -45 | 50 |
|  | R Put | 437 | 0.001 | 5.65 | 30 | -19 | 8 |
|  | L FP | 569 | 0.001 | 5.62 | -26 | 47 | 33 |
|  | L FP |  | 0.010 | 5.23 | -24 | 41 | 26 |
|  | L HG | 974 | 0.001 | 5.62 | -53 | -21 | 9 |
|  | L STG |  | 0.005 | 5.38 | -54 | -13 | -1 |
|  | L PoG/PCu | 486 | 0.003 | 5.48 | -9 | -40 | 53 |
|  | R PCu | 709 | 0.003 | 5.47 | 21 | -66 | 36 |
|  | R PCu |  | 0.010 | 5.22 | 14 | -61 | 38 |
|  | R PCu |  | 0.012 | 5.18 | 15 | -70 | 40 |

Note: Foci of significant functional connectivity, FWE-corrected for multiple comparisons at a voxel level *p*_FWE_< 0.05, *k*= 250. FWE= Family wise error, k= minimum cluster size in mm^3^, MNI= Montreal Neurological Institute. L=Left, R=Right. Regional names from Harvard-Oxford Cortical and Subcortical Structural Atlas: ACgG=Anterior Cingulate Gyrus, aIns=Anterior Insula, Amg=Amygdala, dCau=dorsal Caudate, vCau=ventral Caudate, FO=Frontal Operculum, FP=Frontal Pole, GP=Globus Pallidus, HG=Heschl's gyrus, NAc=Nucleus Accumbens, PCgG=Posterior Cingulate Gyrus, PCu=Precuneus, pIns=Posterior Insula, PO=Parietal Operculum, PrG=Precentral Gyrus, PoG=Postcentral Gyrus, PT=Planum Temporale, Put=Putamen, STG=Superior Temporal Gyrus, SOC=Superior Occipital Cortex, SPL=Superior Parietal Lobule, Tha=Thalamus, vINS=ventral Insula. Centroid MNI coordinates are listed for each seed from the Scale II Melbourne subcortical atlas.

Supplemental Table 2. Functional Connectivity of the Dorsal Caudate Seeds.

| Seed | Region | Cluster | Peak | | Peak MNI coordinate | | |
| --- | --- | --- | --- | --- | --- | --- | --- |
|  |  | *k* | *p(FWE)* | *Z* | *x(mm)* | *y(mm)* | *z(mm)* |
| **L dCau** | L dCau | 14422 | <0.001 | >8.00 | -11 | 15 | 8 |
| Centroid: [-12,14,7] | L dCau |  | <0.001 | 7.21 | -13 | 2 | 17 |
|  | L Put |  | 0.001 | 6.64 | -23 | 4 | -1 |
|  | L PCG | 11089 | <0.001 | 7.12 | -4 | 19 | 46 |
|  | L PCG |  | 0.001 | 6.55 | -10 | 29 | 33 |
|  | L JPL |  | 0.001 | 6.52 | -5 | 5 | 64 |
|  | R Put | 4625 | <0.001 | 6.86 | 22 | 18 | -1 |
|  | R GP |  | 0.003 | 6.34 | 20 | -1 | 3 |
|  | L dCau |  | 0.018 | 5.89 | 15 | 10 | 14 |
|  | L Put | 618 | 0.012 | 5.99 | -28 | -14 | -1 |
|  | R Cer Crus I | 1052 | <0.001 | 5.84 | 38 | -54 | -34 |
|  | R Cer Crus I |  | 0.001 | 5.66 | 49 | -66 | -30 |
|  | R Cer Crus I |  | 0.033 | 4.95 | 34 | -66 | -27 |
|  | L FP | 771 | 0.001 | 5.72 | -22 | 51 | 33 |
|  | L FP | 376 | 0.002 | 5.53 | -32 | 57 | 16 |
|  | L FP | 279 | 0.016 | 5.11 | -7 | 57 | 10 |
| **R dCau** | R dCau | 20167 | <0.001 | >8.00 | 13 | 16 | 7 |
| Centroid:  [13,14,7] | R OFC |  | <0.001 | 6.58 | 28 | 27 | -7 |
|  | R OFC |  | <0.001 | 6.02 | 13 | 7 | -16 |
|  | L Put | 13962 | <0.001 | 7.49 | -19 | 15 | -4 |
|  | L dCau |  | <0.001 | 7.44 | -16 | 10 | 7 |
|  | L Put |  | <0.001 | 7.19 | -31 | -16 | 9 |
|  | L ACgG | 15759 | <0.001 | 6.70 | -11 | 27 | 32 |
|  | L PCG |  | <0.001 | 6.25 | `-1 | 18 | 45 |
|  | R ACgG |  | <0.001 | 6.20 | 12 | 39 | 4 |
|  | R Tha | 2615 | <0.001 | 6.66 | 2 | -9 | 12 |
|  | L Tha |  | <0.001 | 6.39 | 12 | -20 | 8 |
|  | L Tha |  | 0.001 | 5.64 | 14 | -30 | 14 |
|  | R Cer Lobule V | 311 | <0.001 | 6.11 | 14 | -59 | -15 |
|  | L ACgG | 342 | <0.001 | 6.08 | -6 | -6 | 36 |
|  | R CO | 404 | <0.001 | 6.03 | 42 | -14 | 20 |
|  | R Cer Crus I | 414 | <0.001 | 5.89 | 38 | -54 | -37 |
|  | R Cer Crus I | 307 | 0.002 | 5.54 | 32 | -80 | -32 |
|  | R Cer Crus II |  | 0.036 | 4.92 | 33 | -87 | -38 |
|  | L Cer Crus I | 274 | 0.004 | 5.41 | -49 | -58 | -33 |
|  | L FP | 349 | 0.005 | 5.37 | -25 | 50 | 30 |
|  | L OFC | 282 | 0.006 | 5.32 | -43 | 18 | -9 |

Note: Foci of significant functional connectivity, FWE-corrected for multiple comparisons at a voxel level *p*_FWE_< 0.05, *k*= 250. FWE= Family wise error, k= minimum cluster size in mm^3^, MNI= Montreal Neurological Institute. L=Left, R=Right. Regional names from Harvard-Oxford Cortical and Subcortical Structural Atlas: ACgG=Anterior Cingulate Gyrus, dCau=dorsal Caudate, Cer=Cerebellum, CO=Central Operculum, FP=Frontal Pole, GP=Globus Pallidus, JPL=Juxtapositional Cortex, OFC=Orbitofrontal Cortex, PCG=Paracingulate Gyrus, Put=Putamen, Tha=Thalamus. Centroid MNI coordinates are listed for each caudate seed from the Scale II Melbourne subcortical atlas.

Supplemental Figure 1**.** Functional Connectivity (FC) of the Nucleus Accumbens and Caudate Seeds. Nucleus accumbens (NAc; red), ventral caudate (vCau; yellow), and dorsal caudate (dCAu; green) seed regions were defined using the Melbourne Scale II subcortical atlas. The right and left region labels are: NAc (9, 25), vCAu (10, 26), and dCAu (15, 31). The MNI coordinates indicate location of brain slices and the crosshairs. Presented in neurological orientation.


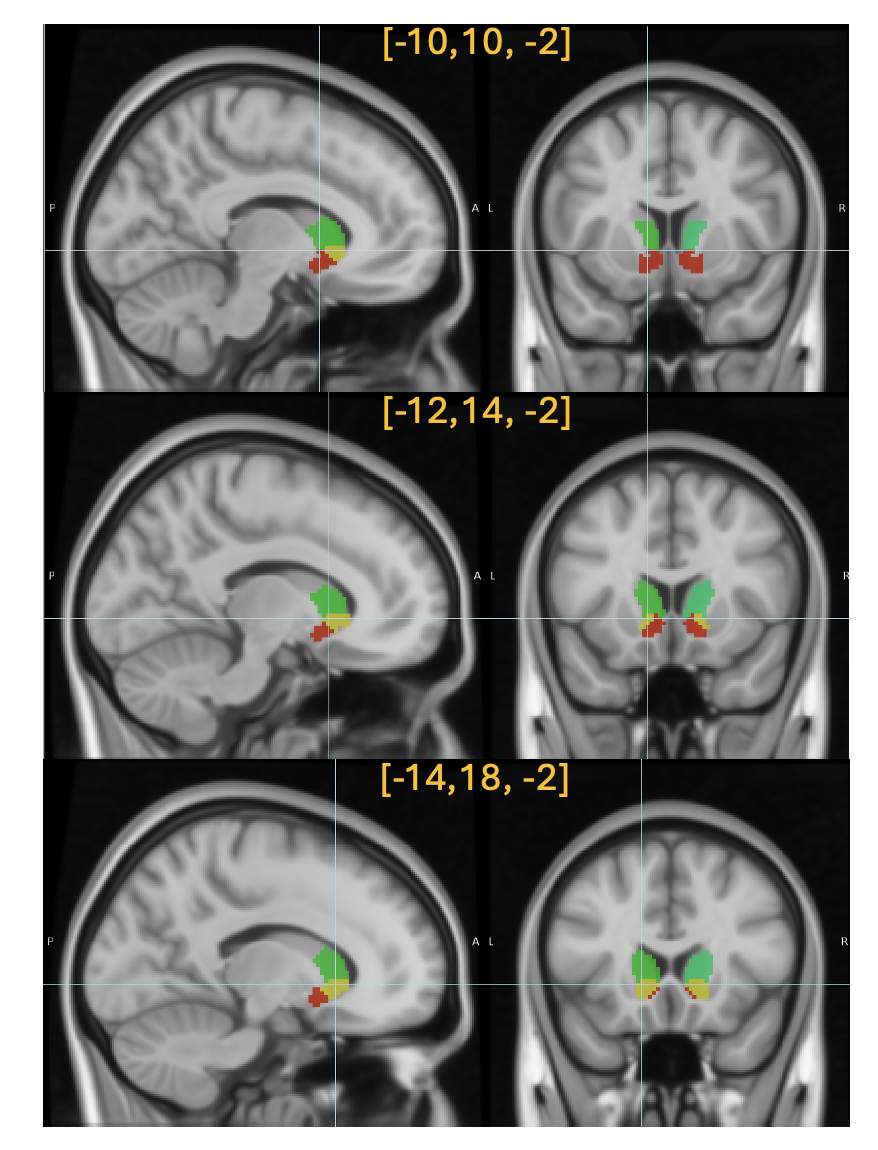


Supplemental Figure 2**.** Functional connectivity (FC) of the Dorsal Caudate (dCau) Seeds; (Left: red-yellow; Right: blue-light blue). Both dCau seeds show prominent connectivity to the contralateral striatum and medial (-12, -6, and 6 sagittal views) premotor and anterior cingulate/prefrontal areas, as well as the cerebellum. The left dCau FC foci are in the left inferior frontal, insula, and frontopolar areas. The right dCau seed also show FC to the insula (x=40) and right orbital areas. Illustrated at voxel-wise *p_FWE_*<0.05, *k*=250). *t*-statistic range in the color bars is 5.34-8. Also see Supplemental Table 2.


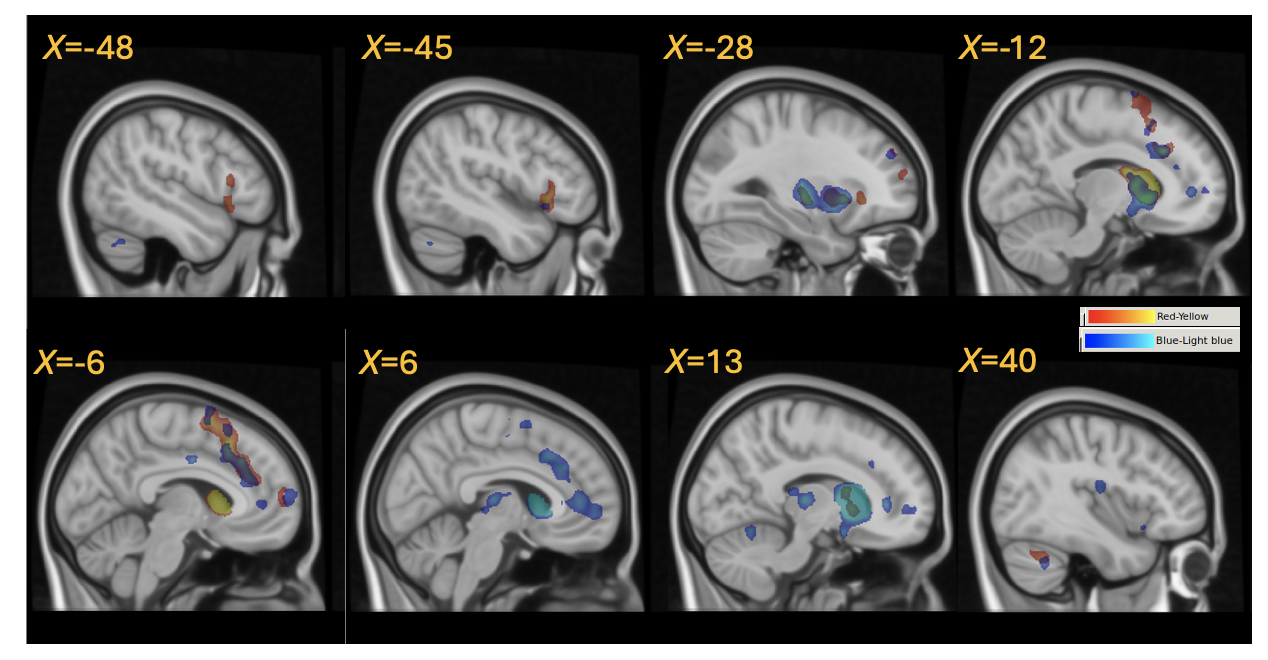

Supplement: Supplementary file 1 — Table S1. Functional connectivity of the NAc and ventral caudate (vCau) seeds. Table S2. Functional connectivity of the dorsal caudate seeds. Figure S1. Functional connectivity (FC) of the nucleus accumbens and caudate seeds. Nucleus accumbens (NAc; red), ventral caudate (vCau; yellow) and dorsal caudate (dCAu; green) seed regions were defined using the Melbourne Scale II subcortical atlas. The right and left region labels are: NAc (9, 25), vCAu (10, 26) and dCAu (15, 31). The MNI coordinates indicate location of brain slices and the crosshairs. Presented in neurological orientation. Figure S2. Functional connectivity (FC) of the dorsal caudate (dCau) seeds; (left: red‐yellow; right: blue‐light blue). Both dCau seeds show prominent connectivity to the contralateral striatum and medial (−12, −6, and 6 sagittal views) premotor and anterior cingulate/prefrontal areas, as well as the cerebellum. The left dCau FC foci are in the left inferior frontal, insula and frontopolar areas. The right dCau seed also show FC to the insula (x = 40) and right orbital areas. Illustrated at voxel‐wise p FWE < 0.05, k = 250). t‐Statistic range in the colour bars is 5.34–8. Also see Table S2. [file EJN-61-0-s001.docx]
